# Quality of Life and Multilevel Contact Network Structures Among Healthy Adults in Taiwan: Online Participatory Cohort Study

**DOI:** 10.2196/23762

**Published:** 2022-01-28

**Authors:** Tso-Jung Yen, Ta-Chien Chan, Yang-Chih Fu, Jing-Shiang Hwang

**Affiliations:** 1 Institute of Statistical Science Academia Sinica Taipei Taiwan; 2 Research Center for Humanities and Social Sciences Academia Sinica Taipei Taiwan; 3 Institute of Sociology Academia Sinica Taipei Taiwan

**Keywords:** contact diary, egocentric networks, social support, weak ties, World Health Organization Quality of Life Survey, quality of life, networks, demography, society

## Abstract

**Background:**

People’s quality of life diverges on their demographics, socioeconomic status, and social connections.

**Objective:**

By taking both demographic and socioeconomic features into account, we investigated how quality of life varied on social networks using data from both longitudinal surveys and contact diaries in a year-long (2015-2016) study.

**Methods:**

Our 4-wave, repeated measures of quality of life followed the brief version of the World Health Organization Quality of Life scale (WHOQOL-BREF). In our regression analysis, we integrated these survey measures with key time-varying and multilevel network indices based on contact diaries.

**Results:**

People’s quality of life may decrease if their daily contacts contain high proportions of weak ties. In addition, people tend to perceive a better quality of life when their daily contacts are face-to-face or initiated by others or when they contact someone who is in a good mood or someone with whom they can discuss important life issues.

**Conclusions:**

Our findings imply that both functional and structural aspects of the social network play important but different roles in shaping people’s quality of life.

## Introduction

People’s quality of life (QoL) diverges not just on where they stand in their life cycle and the socioeconomic hierarchy but also on how they are connected with others. Overall, well-connected persons tend to perceive a better quality of their lives [1-11]. Following various definitions and instruments of social networks, existing studies have verified such positive links between social networks and QoL. For example, the structure of the social network is highly correlated with QoL among female workers with high levels of stress at home and work [12]. It also differs significantly between cancer patients with high QoL and those with low QoL [13]. In addition, QoL of older adults varies significantly by the structure of their social networks in terms of the existence of a spouse, size of their family, contact frequency of family members, and network of friends [14]. Moreover, when patients with mental illness discuss their health issues more often with their social network members, it is more likely that they will recover from the disease [15].

Although these studies make various contributions to the literature, they tend to focus on specific occasions or groups. It is relatively unknown how social networks are associated with QoL in everyday life or among healthy and young adults. In addition, previous studies on QoL tended to measure people’s QoL or social networks based on cross-sectional instruments. When measuring personal networks, some studies are further limited by focusing on network size only. Although network size is an important measure of a social network’s global structure, it reveals little about the social network’s local structure. In other words, network size is insufficient for reflecting how people interact with others in their social networks and how they feel about one another during social interactions. Other than network structures, such functional aspects of social networks are also important for understanding how the social networks are associated with QoL [5].

In this study, we examined how QoL varies in both structure and function of social networks, by analyzing diary data of healthy young adults’ social contacts. We adopted a repeated measurement design and conducted a 4-wave survey to measure participants’ QoL using the brief version of the World Health Organization Quality of Life Survey (WHOQOL-BREF). In addition, we tracked participants’ daily social interactions using in-depth contact diaries [16-18]. We used these diary data to build archives about comprehensive personal networks [19] that reveal participants’ social networking in everyday life. The diary data also allow us to pay attention to the functional aspects of the social networks, especially how people feel about each specific interpersonal contact. By integrating both survey and diary data, we were able to investigate how social networks are associated with QoL at contact, tie, and individual levels. Following this bottom-up approach [20], along with multiple-wave measures and multilevel research designs, we aimed to better capture how social networks shape people’s daily life experiences.

## Methods

### Recruitment and Data Collection

**Figure 1:**
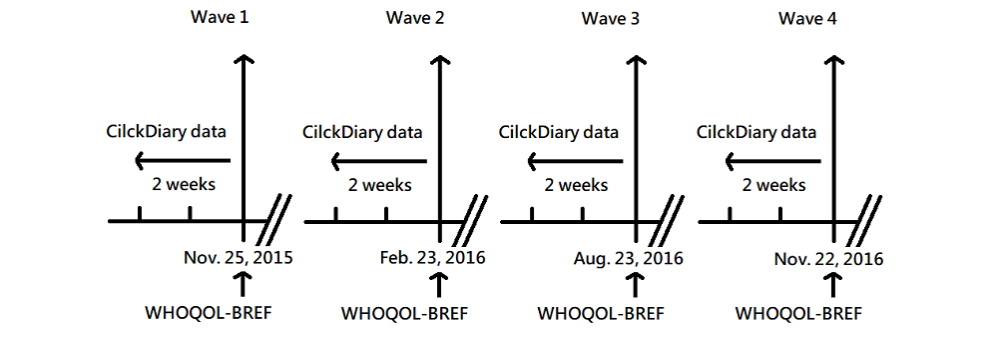
Schematic plot of the data collection procedure. WHOQOL: World Health Organization Quality of Life survey.

We recruited participants and collected their survey and diary data via an online platform called ClickDiary [16,17]. Based on a web-based design, ClickDiary helps researchers implement and manage diary studies and lowers the burden for research participants to record repetitive and unique content relevant to various contact situations. To facilitate compatible analysis, we extracted 2 data sets collected between November 25, 2015 and November 22, 2016. The first data set consisted of a 4-wave survey of participants’ QoL measured using the WHOQOL-BREF [21]. This measurement has been further modified to take local social and cultural norms into account [22,23]. We implemented the 4-wave survey on November 25, 2015; February 23, 2016; August 23, 2016; and November 22, 2016. Figure 1 shows a schematic plot of the data collection procedure. In our analysis, we only focused on healthy participants. We excluded from our data set participants who had at least one of the following chronic diseases: cardiovascular disease, diabetes, and cancer. The reason we excluded these participants is that, although it is well-known that these chronic diseases play important roles in shaping people’s QoL, among 154 participants in our original data set, only 5 participants had these chronic diseases. The sample size was not large enough to validate the importance of these chronic diseases on people’s QoL. After excluding the 5 participants, all remaining 149 participants in our data set did not have these chronic diseases. Among the participants, 34 completed the survey in all 4 waves, 51 completed 3 waves, 28 completed 2 waves, and 36 completed only 1 of the 4 waves. In total, the participants generated 358 records of the survey. The participants in our sample were mainly young adults with an average age of 36 years. Of 149 participants, 110 (73.8%) were female. Being skewed in terms of age and gender, a typical bias present in in-depth diary studies [19], our sample was not representative by any means, and we thus refrained from making any inference from our findings about survey data. Instead, we focused on how the survey results could benefit from detailed information in the diary data. The statistics on age and gender, along with other personal attributes, are summarized in Table 1.

The second data set consisted of detailed contact records nested at 3 unique, yet intertwined, levels taken from ClickDiary: individual characteristics of the participant (that is, the diary keeper, or “ego” in the participant’s egocentric network), unique attributes of the contact person (“alter”) or the relationship between ego and alter (“tie”), and the situation of each specific contact between each ego-alter pair (“contact”). Each participant recorded contact information and health information in ClickDiary on a daily basis. To better align such information with the survey measures, we constructed social network attributes using only the contact information the participants recorded 2 weeks before they completed a wave of the WHOQOL-BREF survey. These social network attributes in turn facilitated the analysis of how they could help determine QoL.

Because a typical in-depth contact diary study requires its participants to devote themselves to recording interpersonal contacts in all forms for months, and sometimes over a year, the task of diary-keeping has been so demanding and tedious that only a small group of volunteers could possibly finish the study even with reasonable incentive and monetary compensation [19]. As a result, diary studies are extremely difficult to implement, and only a handful of diary data sets has been available in the past decade [16-18]. Unlike other diary data sets, the data set we used for analysis integrated the aforementioned standardized QoL measurements in multiple waves, which appropriately facilitated our research goal.

**Table 1:** Summary statistics of the ego-level attributes (n=149).

Variable	Results
Male gender, n (%)	39 (26.2)
Male gender, SD	44
Age (years), mean (SD)	35.9 (12.6)
Education^a^, mean (SD)	3.99 (0.5)
Nonneurotic personality^b^, mean (SD)	1.85 (0.71)
Exercise (hours), mean (SD)	0.27 (0.31)
Sleep well, n (%)	61 (40.9)
Sleep well, SD	49.2
BMI 21-24 kg/m^2^, n (%)	52 (34.9)
BMI 21-24 kg/m^2^, SD	47.7
Network size (log scale), mean (SD)	4.26 (0.71)

^a^Education was coded in 6 levels as follows: 0 = no education, 1 = elementary school, 2 = middle school, 3 = high school, 4 = college/university, and 5 = graduate school.

^b^Nonneurotic personality was measured with a scale ranging from 0 (disagree strongly) to 3 (agree strongly).

### Response Variables

The WHOQOL-100 survey was developed through a collaboration of 15 sites around the world using a common protocol with different languages [24]. Each of the 100 items in this original scale contains responses on a 5-point Likert interval. To simplify the instrument, the WHOQOL-BREF [22] was developed. The WHOQOL-BREF consists of only 26 crosscultural items spanning 4 dimensions of QoL: physical, psychological, social, and environmental. With excellent reliability, this 26-item scale measures people’s QoL across different cultural and societal settings [25,26]. In addition to the 26 items, 2 extra items are added to represent unique cultural attributes relevant to the QoL of Taiwanese people. The first concerns personal respectfulness, which belongs to the social dimension. The second focuses on the availability of food, part of the environmental dimension. From the 4-wave WHOQOL-BREF survey, we calculated scores for the physical, psychological, social, and environmental dimensions as follows. First, we divided the 28 (26 plus 2) items in WHOQOL-BREF into the 4 dimensions according to the WHOQOL-BREF guidelines [22]. To make the 4 scores comparable, we further rescaled the scores into a range between 0 and 100. We also calculated a summary score by summing the rescaled 4-dimensional scores. This summary score was further rescaled into a range between 0 and 100 for measuring a participant’s overall QoL [22].

**Figure 2:**
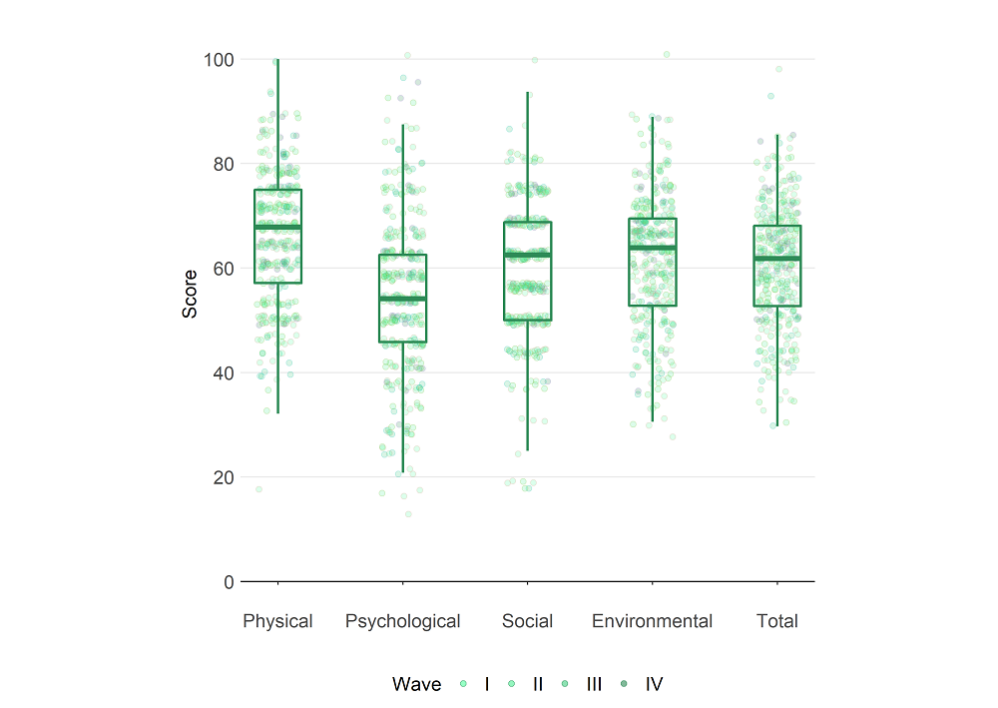
Boxplot of quality of life scores, as calculated using the guideline described in the World Health Organization Quality of Life (WHOQOL) Taiwan Version [22]: physical, psychological, social, environmental, and corresponding total scores.

Figure 2 shows a boxplot of the 4 waves of the QoL scores for physical, psychological, social, environmental, and overall dimensions of the participants. The means (SDs) of the 5 QoL scores were 66.0 (12.4), 54.7 (15.5), 59.1 (12.7), 62.1 (12.4), and 60.5 (11.4), respectively.

### Contact Data

The ClickDiary platform is a simplified version of a contact diary that aims to reach more volunteer participants by reducing the burden of diary keeping while retaining the core elements of contact diaries. It allows participants to report information about daily contacts via a website with a responsive design adjusted for different electronic devices. Unlike conventional means of network data collection, ClickDiary contributes to the methodology with 2 distinct features. First, the platform is cost-effective since it pays exclusive attention to personal network information and does not require knowledge of the whole network [27]. Second, the diary design helps generate network data on a daily basis. The contact networks based on such diary data follow a longitudinal format that can yield more continuous information. This differs from both the network data collected via one-shot surveys and the unstructured contact records collected from social media.

By tracing all kinds of contact between ego and alters, ClickDiary probes the type, time, location, and duration of each contact. It also asks the participant to report the consequences of the contact, such as the extent to which each specific contact benefits ego and leaves ego in a good mood after the contact. Besides personal information about ego, ClickDiary also requires the participant to provide detailed information about each alter’s demographic and socioeconomic background. Each participant further judges the strength of all ego-alter ties and estimates the strength of all alter-alter ties within each egocentric network. This further enables us to investigate the impact of the social network structure on a particular contact outcome. With a large number of alters, reporting this kind of information may take a long time and can be laboriously intensive, and some commitment is required to accomplish the job. To enhance this commitment, we designed a monetary incentive to encourage participants to report responsibly and correctly. The research team checked the data quality each week and set up rules to exclude unreliable data and participants.

Data collected via ClickDiary are organized in a hierarchical structure that involves both space-time and interpersonal dimensions. This hierarchical structure contains 3 levels: (1) ego level, at which the participant (ego) is identified; (2) alter level, at which an alter of the participant (ego) is identified; (3) contact level, at which a daily contact between the participant and an alter is identified. In our study, we explored the hierarchical structure to calculate the attributes of each ego and the attributes of each egocentric network. In other words, we used the data collected at the ego level to calculate personal attributes for the participant, and the data collected at the alter and contact levels were used to calculate the attributes of the social network surrounding the participant. In addition, we summarized each network attribute in a propensity score ranging between 0 and 1, which in turn served as a covariate (independent variable) in the subsequent regression analysis on how QoL is linked with social networks. Because we asked the participants about their QoL conditions during the previous 2 weeks, we calculated the propensity scores using only data collected 2 weeks before a wave of the WHOQOL-BREF survey was conducted. In particular, the propensity score of a social network attribute was calculated by aggregating the frequency of the contacts that matched this attribute during the previous 2 weeks.

### Independent Variables

The independent variables in our analysis cover ego’s personal attributes and the attributes of the social network surrounding each ego. We utilized the following 8 personal attributes for each ego: gender, age, education, nonneurotic personality, daily exercise time, sleep quality, BMI, and personal network size (Table 1).

We used 5 social network attributes at the alter level for each ego. All 5 network attributes were summarized as propensity scores that were calculated using diary data collected during the 2 weeks before we queried respondents using the WHOQOL-BREF scale. The 5 attributes were the proportion of alters who were strongly tied with ego (strong tie); proportion of alters with whom ego could discuss important issues (important issue); proportion of alters of the same gender as ego (gender homophily); proportion of alters in the same age cohort as ego (age homophily); and averaged embeddedness score of the alters (embeddedness, or the extent to which alters knew one another within each egocentric network).

**Figure 3:**
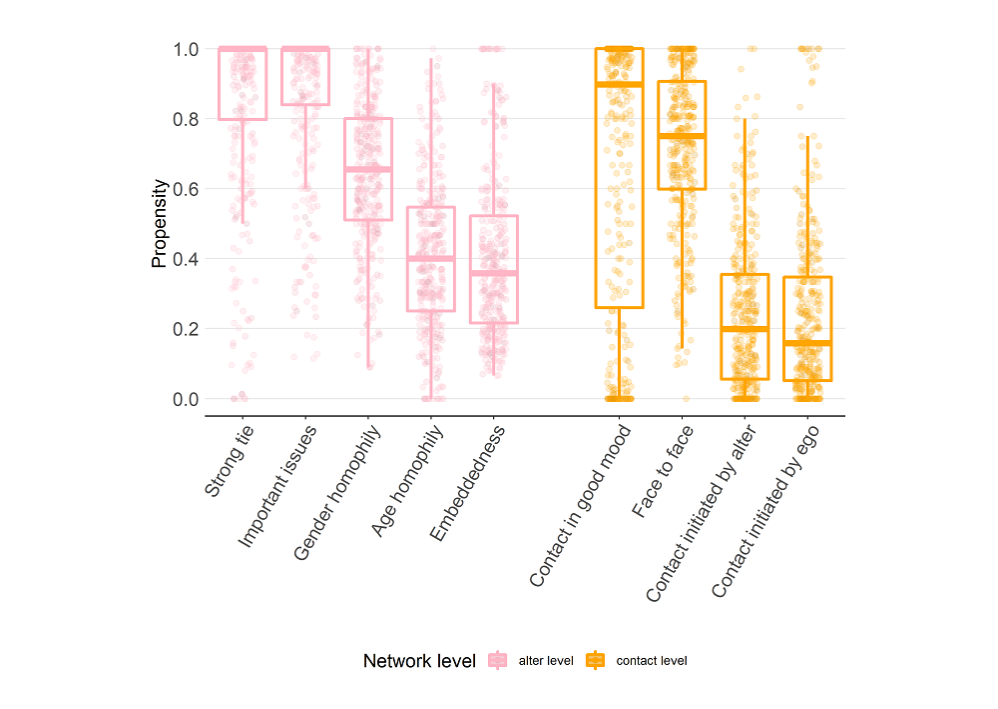
Boxplot of the alter-level attributes (pink) and contact-level attributes (orange).

We further reconstructed 4 social network attributes for each ego from data at the contact level: proportion of the contacts in which ego felt that the alters were in a good mood, proportion of contacts that were conducted face-to-face, proportion of contacts that were initiated by the alters, and proportion of contacts that were initiated by ego. Figure 3 presents a boxplot of the 5 alter-level attributes and 4 contact-level attributes. Because these attributes are defined in terms of proportion, their values are between 0 and 1.

### Statistical Analysis

**Figure 4:**
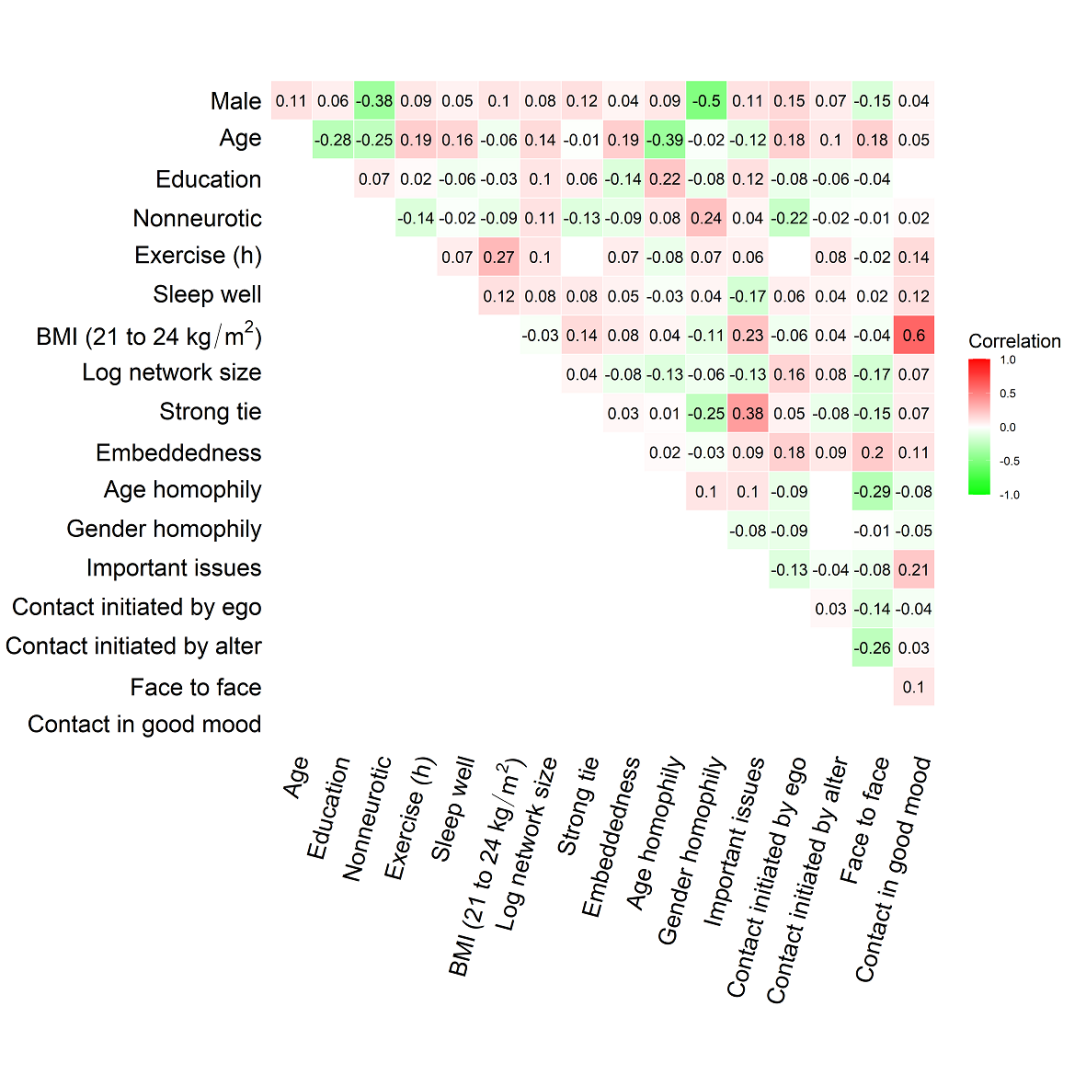
Correlation plot of the ego-level, alter-level, and contact-level attributes.

**Figure 5:**
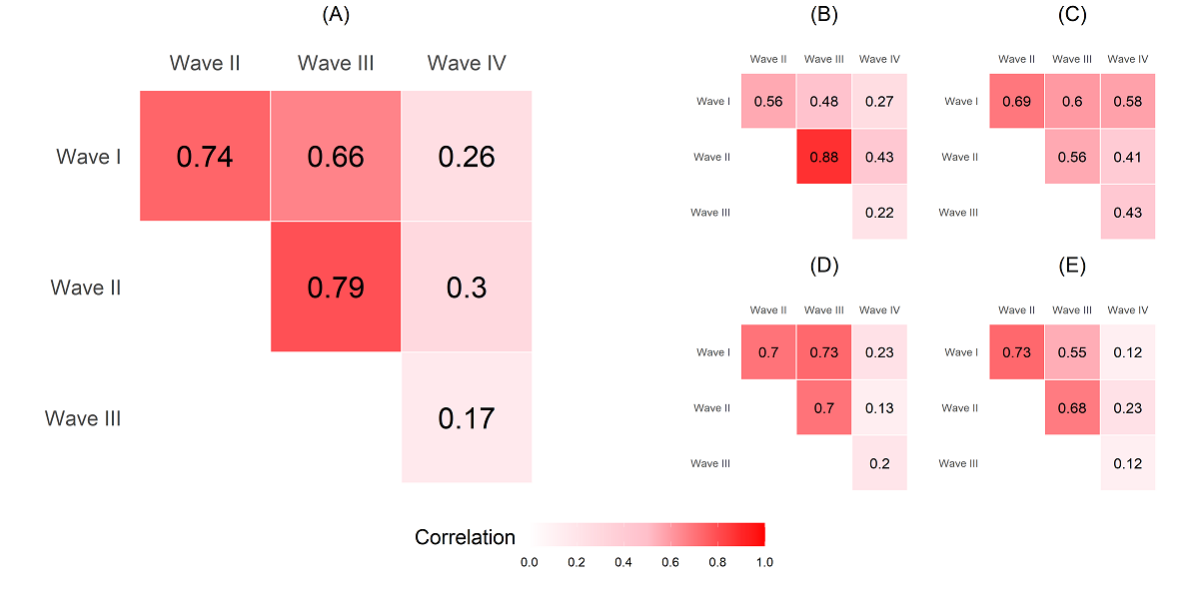
Correlation plot of the responses in the 4-wave surveys: (A) total score, (B) physical score, (C) psychological score, (D) social score, (E) environmental score.

We investigated how QoL is linked to social networks by conducting statistical analysis using the 4-dimension scores and the total score of QoL as the response variables and the social network attributes as the independent variables. Here, we adopted a regression approach to analyzing the data. The regression approach is an effective way to investigate the relationship between a response variable and a set of independent variables. As we are interested in the relationship between QoL and the social networks, a regression model can help us to clarify what aspects of the social networks are associated with QoL. It can also control the variables that may exhibit confounding effects on the response. However, to conduct a proper regression analysis, we needed to pay attention to possible correlations among the independent variables. Figure 4 shows a plot of correlations of the independent variables: 8 ego-level attributes, 5 alter-level attributes, and 4 contact-level attributes. Because the correlations among these attributes were not high, these attributes were appropriate as independent variables in the regression analysis. The weak correlations among the ego-level, alter-level, and contact-level attributes also allowed us to use a linear regression model to examine the relationship between the response variable (QoL measure) and independent variables (the attributes) since we did not need to consider collinearity among the independent variables. Collinearity among independent variables will destabilize computation of the Gram matrix of independent variables. It will increase the variance of the estimated regression coefficients, further distorting interpretation of the results provided by the regression analysis. On the other hand, because the response variable was measured repeatedly, the measured scores of the consecutive waves tended to correlate with each other. Figure 5 shows correlation plots of the 4-dimension scores and the total QoL score in the 4-wave survey (labeled I, II, III, and IV). These plots indicate that the scores of consecutive waves were highly correlated. These high correlations may have had a serious impact on regression model estimation, particularly on the variance of the estimated regression coefficients. To address this problem, we adopted the generalized estimating equation (GEE) method to estimate the regression models [28]. The GEE method takes the correlation structure of the response into account when estimating the coefficients of the regression models.

## Results

**Figure 6:**
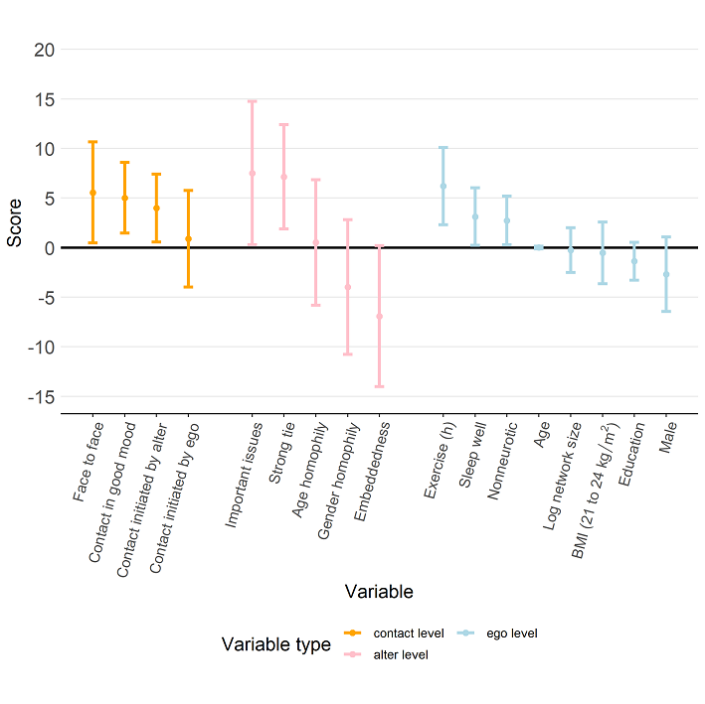
Estimation results from the regression model with the total score as the response (dependent variable). The estimated regression coefficients corresponding to the contact-level attributes, alter-level attributes, and ego-level attributes are shown in orange, pink, and light blue, respectively, and the bars represent the 95% CI for the estimated regression coefficient.

We estimated the regression models separately for each of the 4 dimensions of QoL scores and the total score. Figure 6 shows the estimation results for the model with the total score as the response variable. The results revealed that a participant’s self-reported total score of QoL was higher when the daily contacts consisted of a larger proportion of alters who were strongly tied (estimated regression coefficient 7.15, 95% CI 1.88 to 12.42), who could discuss important issues with the participant (coefficient 7.52, 95% CI 0.30 to 14.75), or who were in a good mood (coefficient 5.02, 95% CI 1.45 to 8.58) as well as contact via face-to-face meetings (coefficient 5.57, 95% CI 0.46 to 10.67). In addition, total QoL score was higher if contact was initiated by alters (coefficient 3.99, 95% CI 7.42 to 0.57). On the other hand, we also found that the size of the social network surrounding a participant did not have an impact on the participant’s total score (coefficient –0.241, 95% CI –2.50 to 2.02). Although contacting a high proportion of alters who are embedded in a participant’s personal network may have a negative impact on the participant’s total QoL score, such a structural factor was not statistically significant (coefficient –6.91, 95% CI –14.02 to 0.20).

**Figure 7:**
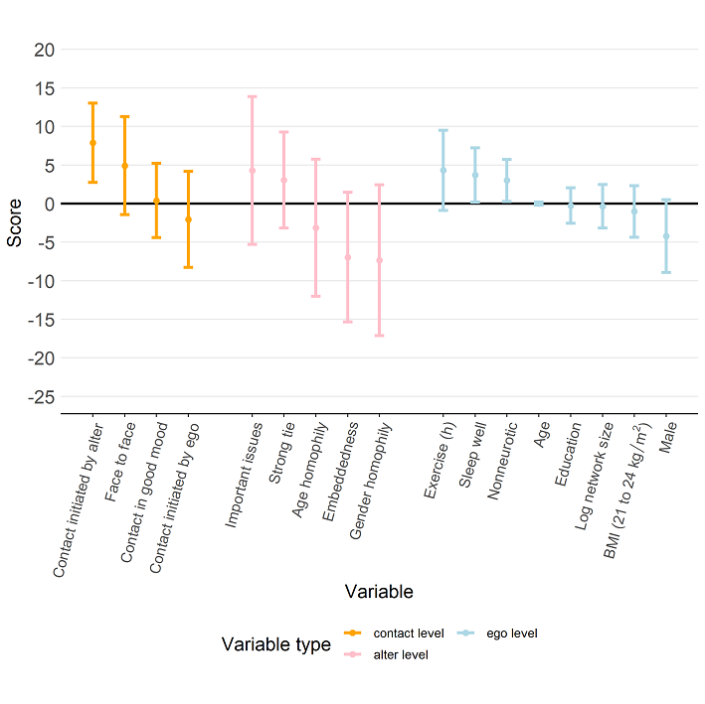
Estimation results from the regression model with the physical score as the response (dependent variable). The estimated regression coefficients corresponding to the contact-level attributes, alter-level attributes, and ego-level attributes are shown in orange, pink, and light blue, respectively, and the bars represent the 95% CI for the estimated regression coefficient.

**Figure 8:**
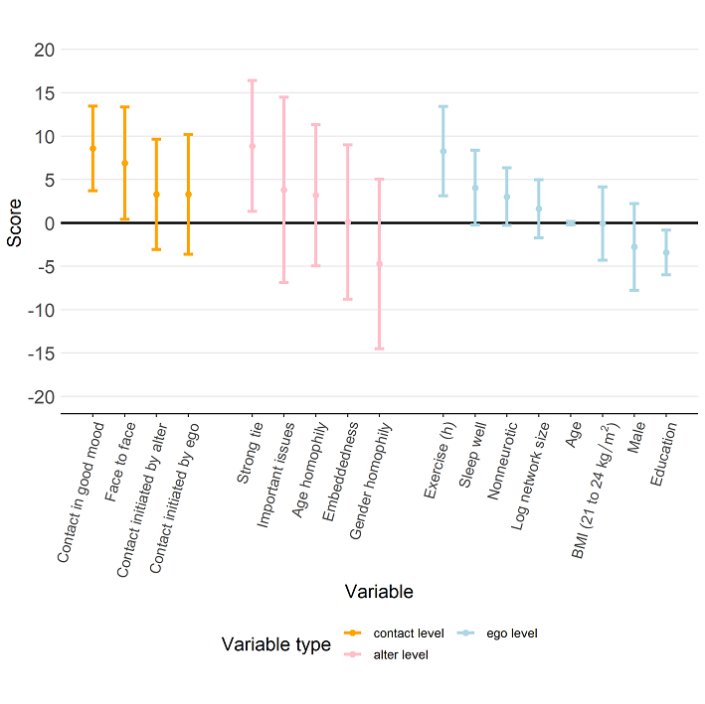
Estimation results from the regression model with the psychological score as the response (dependent variable). The estimated regression coefficients corresponding to the contact-level attributes, alter-level attributes, and ego-level attributes are shown in orange, pink, and light blue, respectively, and the bars represent the 95% CI for the estimated regression coefficient.

**Figure 9:**
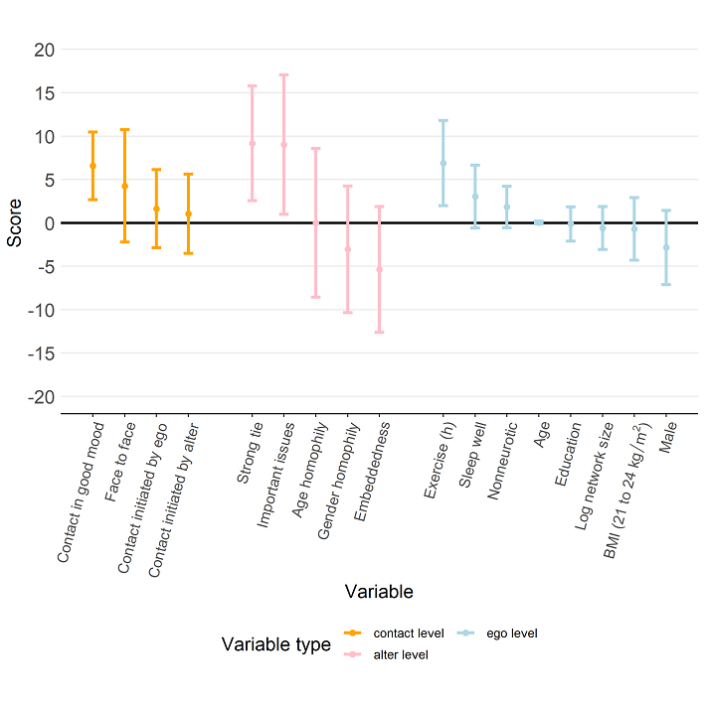
Estimation results from the regression model with the social score as the response (dependent variable). The estimated regression coefficients corresponding to the contact-level attributes, alter-level attributes, and ego-level attributes are shown in orange, pink, and light blue, respectively, and the bars represent the 95% CI for the estimated regression coefficient.

**Figure 10:**
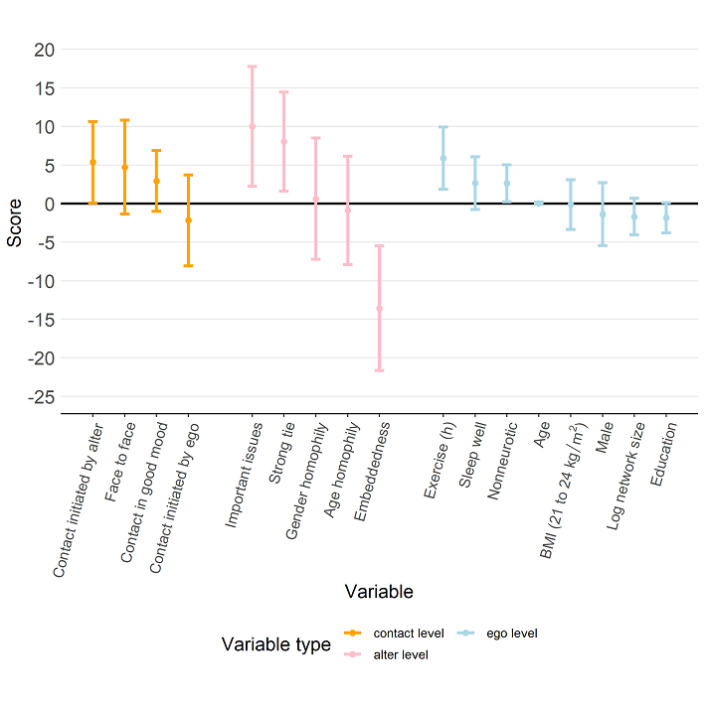
Estimation results from the regression model with the environmental score as the response (dependent variable). The estimated regression coefficients corresponding to the contact-level attributes, alter-level attributes, and ego-level attributes are shown in orange, pink, and light blue, respectively, and the bars represent the 95% CI for the estimated regression coefficient.

Figure 7 shows that a participant’s physical QoL score was higher when more contact was initiated by alters (coefficient 7.88, 95% CI 2.75 to 13.01). Figure 8 further indicates that a participant’s psychological QoL score was positively associated with the proportion of contact that was face-to-face (coefficient 6.88, 95% CI 0.40 to 13.36) and contact during which the alter was in a good mood (coefficient 8.59, 95% CI 3.71 to 8.59). Psychological QoL also seemed to be better with a higher proportion of contact in which the alter was strongly tied to the participant (coefficient 8.86, 95% CI 1.33 to 16.39). Figure 9 shows that a participant’s social QoL score was positively associated with the proportion of contact in which the alter was in a good mood (coefficient 6.57, 95% CI 2.68 to 10.46), the alter could discuss important issues (coefficient 9.02, 95% CI 0.98 to 17.05), and the alter was strongly tied with the participant (coefficient 9.17; 95% CI 2.56 to 15.78). Figure 10 suggests that a participant’s environmental score was positively associated with the proportion of alters who could discuss important issues (coefficient 10.01, 95% CI 2.23 to 17.79) and the proportion of alters who were strongly tied with the participant (coefficient 8.02, 95% CI 1.60 to 14.44) but negatively associated with the embeddedness score (coefficient –13.58, 95% CI –21.66 to –5.50).

## Discussion

In this paper, we investigated the relationships between QoL and social networks by jointly analyzing 2 sets of online platform data collected via longitudinal surveys and contact diaries. According to the regression analysis, the overall QoL among these healthy young adults tended to be higher when they contacted those who were connected well with them, who could discuss important issues with them, and who made them feel better during an interaction. These findings imply that both functional and structural aspects of social networks play important roles in shaping one’s QoL. The participants tended to benefit by interacting with people they knew well, who were trustworthy, and who were in a positive mood. Overall QoL was also more likely to increase if more of their daily contacts were initiated by the other party. This interesting finding may be surprising because earlier diary studies indicated that a person tends to benefit from a specific social exchange while initiating a contact [29]. Overall, however, constantly reaching out to others may not bring one the best QoL in the long run. Being approached or “invited” by others, after all, may signal a form of “respectfulness” embedded in social relationships that helps boost one’s perception of QoL.

In contrast, network size alone was not significantly linked to overall QoL, after taking both the functional and structural aspects of social networks into account. This finding is not surprising because network size was used as a proxy measure for functional and structural aspects of social networks. Once the functional and structural aspects of the social network are both incorporated into the model, the effect of network size disappears.

More specifically, the functional and structural aspects of social networks may play various roles in shaping different dimensions of QoL. For example, the participants’ physical QoL increased when their daily contacts were frequently initiated by other people, even though this factor did not help other dimensions of QoL. In addition to the possible “respectfulness” suggested in the previous paragraph, this finding also may have to do with one’s physical condition: Better physical health tends to facilitate or attract more invitations, or initiations, from social contacts. Moreover, the psychological and social dimensions of people’s QoL may get better if they frequently interact with those who have positive moods. However, this “positive energy” factor does not have a significant impact on helping the physical and environmental dimensions of QoL.

There are noted limitations to this study. Although our data follow a longitudinal format, the results cannot be used to verify the causal relationships between social networks and QoL. In addition, our results are based on information about the social network surrounding the participant, which has been constructed through contact diaries repeatedly recorded by the participant. Although this diary approach tends to yield active and comprehensive archives of personal networks and is thus more stable and reliable than one-shot survey data, these self-reported contact records may not be free from recall biases. It is thus difficult to rule out potential measurement errors, even though we tried to verify any doubtful information by calling participants to double check and correct the data.

Our data have a repeated but “imbalanced” measurement format in which participants did not have equal observations, which may be another concern for statistical analysis. Because the social network effects we were interested in were neither individual-specific nor time-specific, we could still estimate our regression models by treating the data as repeated measurement data without a strong assumption on the missing mechanism of the unobserved data points. What we needed to pay attention to was the within-subject correlations between each measurement, which were high in our case, as shown in Figure 6. We tackled this issue by estimating our regression model with the GEE method, a method developed for dealing with data in which observations are dependent.

In our regression analysis, we treated only social network attributes as the main factors. Such network effects may interact with demographic and socioeconomic factors; recent studies have found that social networks play an important role in mediating the impacts of gender [30,31], age [32], and ethnicity [33] on health-related QoL. Not only does the social network function as a mediator but it may also reciprocally influence other factors in their impacts on QoL [34]. To take these possible multiple roles into account, future studies may want to examine the circumstances under which the social network attributes mediate the sociodemographic covariates that control the paths between other factors and QoL.

## Abbreviations

GEE: generalized estimating equation
QoL: quality of life
WHOQOL: World Health Organization Quality of Life
